# Leukocytosis in Diabetic Ketoacidosis Without Infection: A Diagnostic Challenge

**DOI:** 10.7759/cureus.107902

**Published:** 2026-04-28

**Authors:** Khadija Ali, Nethmi Chandrasekara, Man Wai Cheng, Hemantha Chandrasekara

**Affiliations:** 1 Emergency Medicine, North Manchester General Hospital, Manchester, GBR; 2 Internal Medicine, Manchester University NHS Foundation Trust, Manchester, GBR; 3 General Medicine, North Manchester General Hospital, Manchester, GBR; 4 Diabetes and Endocrinology, Manchester University NHS Foundation Trust, Manchester, GBR

**Keywords:** diabetes, diabetic ketoacidosis, emergency, infection, leukocytosis

## Abstract

Diabetic ketoacidosis (DKA) is a potentially life-threatening condition that requires prompt and precise management. While concurrent infection is a common precipitating factor, an elevated white blood cell (WBC) count does not necessarily indicate active infection.

This case report illustrates a patient presenting with DKA and a high WBC count, but not showing any signs of infection. WBC level normalised after treatment of DKA, without the use of antibiotics. C-reactive protein and procalcitonin levels aided decision-making. Further studies are needed to validate the diagnostic value of these biomarkers and potentially establish a reliable tool to guide antibiotic stewardship. Reducing unnecessary antibiotic use in this context may significantly lower hospital costs and mitigate the growing threat of antimicrobial resistance. Optimising infection assessment in DKA patients remains an important clinical and research priority.

## Introduction

Diabetic ketoacidosis (DKA) is a life-threatening acute complication of diabetes mellitus characterised by hyperglycaemia, metabolic acidosis and ketonaemia [1]. It results from absolute or relative insulin deficiency combined with an increase in counter-regulatory hormones such as glucagon, cortisol, growth hormone and catecholamines [1]. These hormonal shifts lead to enhanced lipolysis and ketogenesis, causing metabolic derangements that require urgent medical attention.

While DKA is more frequently associated with type 1 diabetes, triggered by non-adherence to therapy, new diagnosis, or infection, it is also seen in patients with type 2 diabetes [1,2]. Other recognised triggers include infection, myocardial infarction, stroke, pancreatitis, trauma, surgery, alcohol abuse, certain medications [3] (e.g. corticosteroids, antipsychotics, sodium-glucose co-transporter-2 (SGLT-2) inhibitors) and psychological stress. Among these, infection remains the most frequent precipitating factor, particularly in patients with type 2 diabetes mellitus [4-6].

Due to this association, empirical antibiotic therapy is often initiated early in DKA management while the source of infection is being investigated. Leukocytosis, which is often considered a marker of infection, can, however, also occur as part of the systemic inflammatory response intrinsic to DKA [7]. This nonspecific elevation in WBC count can cloud clinical decision-making, potentially leading to unnecessary antibiotic use. Despite this, there remains limited emphasis in the literature on how to distinguish physiological leukocytosis from infection-driven inflammation in the acute setting, and how this distinction should guide antibiotic prescribing.

This case report describes a patient admitted with DKA and a significantly elevated WBC count, but without clinical, microbiological or radiological evidence of infection. The patient was managed without antibiotics, and the leukocytosis resolved following appropriate DKA treatment alone. This case highlights a clinically relevant but under-discussed scenario and aims to underscore the need for more judicious evaluation of leukocytosis in DKA, addressing the risk of unnecessary antimicrobial use when infection cannot be substantiated.

## Case presentation

A man in his 40s, transferred from a psychiatric inpatient unit to the acute medical service, presented with abdominal pain and vomiting for 2 days. He denied fever, cough, sputum production, diarrhoea and dysuria. His past medical history included type 2 diabetes mellitus, previously on metformin and alogliptin. His HbA1c was 102, and he was recently initiated on subcutaneous insulin. He also had a history of bipolar disorder and schizophrenia on valproic acid. According to the psychiatric team, he had refused both food and medications for approximately one week due to ongoing psychiatric symptoms.

The patient appeared drowsy on arrival, but was alert and oriented to time, place and person with a Glasgow Coma Scale of 15. His vital signs were notable for a heart rate of 123 beats per minute and blood pressure of 166/94 mmHg. He was afebrile at 37.0 °C. His respiratory rate was 22, and oxygen saturation was within normal limits on room air. Chest auscultation was clear bilaterally. Abdominal examination revealed a soft, non-tender abdomen without distension or organomegaly. There was no joint swelling, rashes, peripheral oedema or focal neurological deficit.

Initial laboratory investigations demonstrated findings consistent with DKA, with marked hyperglycaemia, high anion gap metabolic acidosis, and positive serum and urinary ketones. Notably, his WBC count was significantly elevated at 24.7 × 10⁹/L and procalcitonin and CRP were low (Table 1). Blood cultures, urine cultures and a viral swab were all negative. Chest X-ray showed no pulmonary infiltrates, and urinalysis was unremarkable for infection. These investigations were done within a few hours of admission. Exclusion of infection was based on the aforementioned investigations and clinical judgment.

**Table 1:** Biochemical results on admission

Parameter	Result	Reference Range
Blood Glucose	26 mmol/L	3.9 – 7.8 mmol/L
Serum Ketones	>7 mmol/L	<0.6 mmol/L
pH (Arterial blood gas)	7.25	7.35 – 7.45
Bicarbonate (HCO₃⁻)	14.3 mmol/L	22 – 26 mmol/L
Lactate	7.3 mmol/L	0.5 – 2.2 mmol/L
White Blood Cell Count	24.7 × 10⁹/L	4.0 – 11.0 × 10⁹/L
C-Reactive Protein	6 mg/L	<10 mg/L
Procalcitonin	0.13 ng/mL	<0.05 ng/mL (normal) <0.5 low risk of sepsis

The patient was promptly managed according to established guidelines for DKA within one hour of admission [8]. Initial management focused on the correction of dehydration and metabolic derangements. He was started on intravenous 0.9% saline for volume resuscitation to address dehydration and improve tissue perfusion. Alongside fluid therapy, a fixed-rate intravenous insulin infusion was initiated to reduce hyperglycaemia and suppress ketogenesis.

Throughout his hospitalisation, the patient’s blood glucose, electrolytes, acid-base status and vital signs were closely monitored, and the patient experienced a single febrile episode. This transient fever resolved quickly following the administration of antipyretic therapy with paracetamol. His symptoms of abdominal pain and vomiting had resolved since admission. Investigations, including negative blood and urine cultures, a negative viral swab and an unremarkable chest imaging, effectively ruled out the suspected sources of infection under consideration. Despite abdominal pain, the patient appeared well, with a soft, non-tender abdomen, no guarding, rigidity, or distension, active bowel sounds, and no palpable masses. In the absence of features of an acute abdomen, abdominal imaging was not indicated. Empiric antibiotics (e.g. co-amoxiclav) were actively considered but withheld due to the absence of a clear focus of infection; this rationale was documented, supporting avoidance of unnecessary exposure and antimicrobial resistance.

The patient’s metabolic parameters gradually improved, with normalisation of blood glucose, acid-base balance, and WBC count (Table 2). Although his CRP was initially markedly elevated, this was considered proportionate to the severity of his vomiting. The insulin infusion was transitioned to subcutaneous insulin therapy. Plans for ongoing diabetes management and psychiatric follow-up were established.

**Table 2:** Biochemical results during admission

Parameter	Day 3	Day 6	Reference Range
Blood Glucose	10.9 mmol/L	6.7 mmol/L	3.9 – 7.8 mmol/L
Serum Ketones	1.1 mmol/L	<0.1 mmol/L	<0.6 mmol/L
pH	7.48	7.46	7.35 – 7.45
Bicarbonate (HCO₃⁻)	25.9 mmol/L	23.5 mmol/L	22 – 26 mmol/L
White Blood Cell Count	12 × 10⁹/L	10.8 × 10⁹/L	4.0 – 11.0 × 10⁹/L
C-Reactive Protein	65 mg/L	3 mg/L	<10 mg/L

## Discussion

DKA is frequently precipitated by infections, often leading clinicians to initiate empirical antibiotic therapy, particularly when leukocytosis is present [2]. Nevertheless, as illustrated in this case and supported by existing literature, elevated WBC counts in DKA may be attributable to the metabolic derangements of DKA itself, rather than an underlying infection.

Xu et al. demonstrated that patients in the DKA group had significantly higher median WBC and neutrophil counts compared to those with diabetic ketosis, stable diabetes and healthy controls [9]. Importantly, these counts were negatively correlated with arterial pH and positively correlated with plasma glucose levels, indicating an association with DKA severity rather than infection. Conversely, lymphocyte counts were decreased in these patients. Another study proposed the neutrophil-to-lymphocyte ratio (NLR) as a predictor of DKA occurrence in the absence of infection [7]. Serum NLR was elevated in DKA patients compared to controls, and increased with the severity of DKA in type 1 diabetes mellitus. It has been suggested that insulin deficiency promotes neutrophil production in the bone marrow and that the stress response involving secretion of adrenaline, cortisol, and inflammatory mediators further augments leukocytosis [7,10].

In addition to WBC counts, inflammatory markers, such as CRP and procalcitonin, were used in this case to assist in distinguishing infectious from non-infectious causes. However, literature indicates that these markers may also be elevated in the absence of infection in DKA. Dalton et al. reported elevated CRP levels in children with DKA, likely reflecting a pro-inflammatory cytokine response rather than infection [11]. Similarly, there are case reports illustrating high procalcitonin levels in DKA patients without evidence of infection [12,13]. Despite this, procalcitonin remains a valuable negative predictor of infection, with some studies reporting a negative predictive value of 91.6% [14].

Accurate identification of patients with leukocytosis secondary to DKA, rather than infection, has important clinical implications. It may reduce unnecessary antibiotic use, minimising the risk of antibiotic resistance and lowering healthcare costs associated with prolonged hospitalisation and medication use. Despite this, further research is required to clarify the relationship between inflammatory markers and infection in DKA, and to establish reliable biomarkers that can guide antibiotic stewardship in this population.

**Figure 1:**
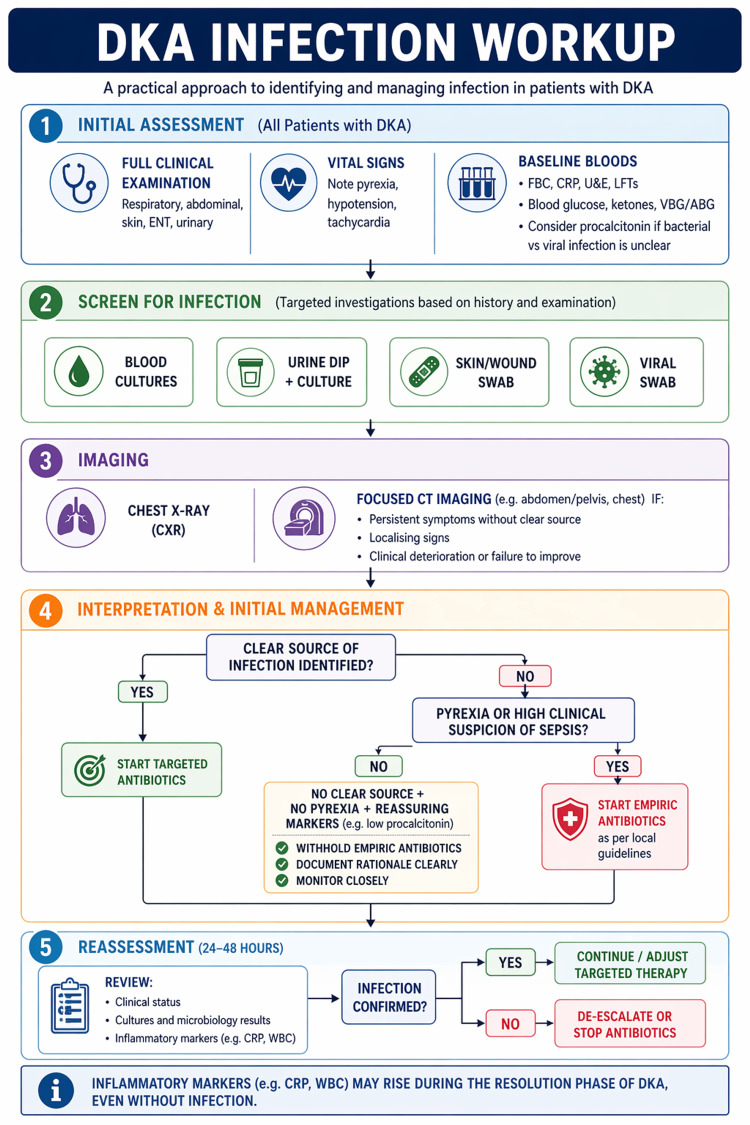
DKA infection workup DKA: diabetic ketoacidosis Image credit: Khadija Ali, using Canva (Canva Pty Ltd, Sydney, Australia)

We have proposed a diagnostic algorithm (Figure 1) for a DKA infection workup, which provides a coherent clinical framework with which clinicians can work. This algorithm can help to facilitate clinicians in identifying the source of infection which may have precipitated DKA, while advocating antimicrobial stewardship and recognising that inflammatory markers may rise later during DKA resolution. 

## Conclusions

In conclusion, this case illustrates that marked leukocytosis can occur in DKA in the absence of an identifiable infection and may resolve with standard metabolic management alone. It highlights the diagnostic challenge of distinguishing stress-related inflammatory responses from true infection in the acute setting. While adjunctive markers, such as CRP and procalcitonin, may support clinical assessment, their interpretation should be cautious and considered alongside the overall clinical picture. This led to a proposed diagnostic workup for DKA infection. However, this report underscores the need for further research to better characterise inflammatory responses in DKA and to clarify their role, if any, in informing decisions around antimicrobial use.

## References

[1] Lizzo JM, Goyal A, Gupta V (2023). Adult diabetic ketoacidosis. StatPearls [Internet].

[2] Feingold KR, Gosmanov AR, Kitabchi AE (2000). Diabetic Ketoacidosis. https://www.ncbi.nlm.nih.gov/books/NBK279146/.

[3] Stamatiades GA, D'Silva P, Elahee M, Viana GM, Sideri-Gugger A, Majumdar SK (2023). Diabetic ketoacidosis associated with sodium-glucose cotransporter 2 inhibitors: clinical and biochemical characteristics of 29 cases. Int J Endocrinol.

[4] Shahid W, Khan F, Makda A, Kumar V, Memon S, Rizwan A (2020). Diabetic ketoacidosis: clinical characteristics and precipitating factors. Cureus.

[5] Mandeel FH, Radhi HT, Sarwani AA, Alsadah AH (2020). Precipitating factors for the development of diabetic ketoacidosis in a tertiary care hospital in Bahrain. Egypt J Hosp Med.

[6] Naveed D, Bilal N, Nasir B, Lodhi BUR (2009). Precipitating factors of diabetic ketoacidosis in type 2 diabetes mellitus. KUST Med J.

[7] Cheng Y, Yu W, Zhou Y, Zhang T, Chi H, Xu C (2021). Novel predictor of the occurrence of DKA in T1DM patients without infection: a combination of neutrophil/lymphocyte ratio and white blood cells. Open Life Sci.

[8] Dhatariya K (2025). Joint British Diabetes Societies for Inpatient Care. The management of diabetic ketoacidosis in adults: JBDS 02 guidelines. https://abcd.care/sites/default/files/site_uploads/JBDS_Guidelines_Current/JBDS_02_DKA_Guideline_with_QR_code_March_2023.pdf.

[9] Xu W, Wu HF, Ma SG, Bai F, Hu W, Jin Y, Liu H (2013). Correlation between peripheral white blood cell counts and hyperglycemic emergencies. Int J Med Sci.

[10] Flood RG, Chiang VW (2001). Rate and prediction of infection in children with diabetic ketoacidosis. Am J Emerg Med.

[11] Dalton RR, Hoffman WH, Passmore GG, Martin SL (2003). Plasma C-reactive protein levels in severe diabetic ketoacidosis. Ann Clin Lab Sci.

[12] Mohammed B, Dweik A, Al-Jobory O, Mcmaster K (2022). Elevated procalcitonin levels in a patient with diabetic ketoacidosis in the absence of infection. Cureus.

[13] Thiab G, Workman A, Khawaja I (2023). An unrecognized cause of elevated procalcitonin level. Cureus.

[14] Mistry N, Sobolewski K, Brophy A (2022). Accuracy of procalcitonin in identifying patients presenting with diabetic ketoacidosis as a result of an infectious etiology. J Lab Precis Med.

